# The roles of small RNAs in rice-brown planthopper interactions

**DOI:** 10.3389/fpls.2023.1326726

**Published:** 2023-11-23

**Authors:** Shengli Jing, Jingang Xu, Hengmin Tang, Peng Li, Bin Yu, Qingsong Liu

**Affiliations:** College of Life Sciences, Xinyang Normal University, Xinyang, China

**Keywords:** sRNAs, *Oryza sativa*, brown planthopper, RNAi, resistance

## Abstract

Interactions between rice plants (*Oryza sativa* L.) and brown planthoppers (*Nilaparvata lugens* Stål, BPHs) are used as a model system to study the molecular mechanisms underlying plant-insect interactions. Small RNAs (sRNAs) regulate growth, development, immunity, and environmental responses in eukaryotic organisms, including plants and insects. Recent research suggests that sRNAs play significant roles in rice-BPH interactions by mediating post-transcriptional gene silencing. The focus of this review is to explore the roles of sRNAs in rice-BPH interactions and to highlight recent research progress in unraveling the mechanism of cross-kingdom RNA interference (ckRNAi) between host plants and insects and the application of ckRNAi in pest management of crops including rice. The research summarized here will aid in the development of safe and effective BPH control strategies.

## Introduction

Rice (*Oryza sativa* L.) is a globally-important staple food which is susceptible to damage from hundreds of insect herbivores throughout its lifecycle (Du et al., 2020). One of the most destructive of these insect herbivores is the brown planthopper (*Nilaparvata lugens* Stål, BPH), which is responsible for severely reduced rice yields and substantial economic losses each year (Shi et al., 2021; Shi et al., 2023). Once outbreaks, the insects can completely destroy crops, an effect called “hopperburn” (Backus et al., 2005).

Plants have evolved an intricate, double-layered defense system to effectively resist and respond to herbivorous pests. The first layer is referred to as pathogen-associated molecular pattern (PAMP)-triggered immunity (PTI) (Jing et al., 2017). PTI activates downstream defense-related signaling cascades, such as the phytohormone-mediated defense response pathway (Erb and Reymond, 2019; Wang et al., 2023). The second layer is known as effector-triggered immunity (ETI), which is a robust resistance (R) protein-mediated defense response (Jones and Dangl, 2006; Takken and Tameling, 2009; Rodriguez et al., 2017). Recent research suggests that plants respond to herbivory through a series of defense-related processes, including phytohormone signaling and secondary metabolite biosynthesis, many of which are regulated by small RNAs (sRNAs) (Sattar and Thompson, 2016).

sRNAs are eukaryotic non-coding RNA molecules, approximately 20-30 nucleotides (nt) in length, which regulate gene expression via RNA silencing (Zamore and Haley, 2005; Chapman and Carrington, 2007). According to their precursor structures and associated genetic pathways, plant sRNAs are classified into two major classes: microRNAs (miRNAs) and small interfering RNAs (siRNAs) (Bartel, 2009; Chen, 2009; Katiyar-Agarwal and Jin, 2010). Likewise, insect sRNAs are divided into three major classes: miRNAs, endogenous-siRNAs (endo-siRNAs), and piwi-interacting RNAs (piRNAs) (Golden et al., 2008). In both plants and animals, miRNAs are 20-24 nt single-stranded non-coding RNAs which mediate post-transcriptional gene silencing by binding to mRNAs containing specific complementary base pairs (Zhang et al., 2006; Bartel, 2009; Ghini et al., 2018). Global sRNA sequence profiling of rice and BPH has enabled the identification and characterization of many sRNAs, particularly miRNAs, involved in rice-BPH interactions (Zha et al., 2016; Wu et al., 2017; Nanda et al., 2020). The focus of this review is to explore the roles of sRNAs in rice-BPH interactions and to highlight recent research progress in unraveling the mechanism of cross-kingdom RNA interference (RNAi) between host plants and insects. The research summarized here will aid in the development of safe and effective BPH control strategies.

## Rice-derived sRNAs involved in BPH resistance

In plants, sRNAs play significant roles in growth, development, abiotic and biotic stress responses (Khraiwesh et al., 2012; Duan et al., 2015; Yue et al., 2017; Chen et al., 2019; Kryovrysanaki et al., 2022). Several studies have utilized RNA and sRNA profiling to identify sRNAs in rice. Functional validation experiments indicate that these sRNAs fine-tune plant innate immunity by integrating *R* gene-mediated resistance, phytohormone signaling, callose deposition, reactive oxygen species (ROS) production, and secondary metabolite biosynthesis (Wu et al., 2017; Ge et al., 2018; Dai et al., 2019; Tan et al., 2020; Lü et al., 2022; Shen et al., 2023).

To date, approximately 17 BPH-resistance (*R*) genes have been identified in both wild and cultivated rice (Wang et al., 2023). Considerable research has been conducted to characterize the mechanism by which *R* genes confer BPH resistance (Jing et al., 2017; Zheng et al., 2021). Through miRNA sequencing, Wu et al. (2017) identified 23 and 674 differentially expressed miRNAs (DEMs) (including 464 known and 183 novel miRNAs) between resistant (carrying BPH-resistance gene *Bph15*) and susceptible rice varieties before and after BPH infestation, respectively. The identified DEMs were primarily involved in basal defense and BPH-specific resistance. Similarly, an integrated miRNA and mRNA analysis identified 217 DEMs between *Bph6*-carrying transgenic rice lines and wild type plants after BPH infestation (Tan et al., 2020). Of these, nine miRNAs were specifically expressed in transgenic rice lines, suggesting their involvement in *Bph6*-mediated resistance to the BPH. In addition, both Nanda et al. (2020) and Lü et al. (2022) identified an array of BPH-responsive miRNAs between resistant and susceptible rice varieties. Although these findings suggest that miRNAs likely participate in the BPH defense response, the involvement of only a few miRNAs has been experimentally verified (**Table 1**).

**Table 1:** The sRNAs involved in rice-BPH interactions.

miRNA	Origin	Target	Acquire method	Reference
*Osa-miR156*	*Oryza sativa*	*Squamosa promoter binding protein-like gene3/11/12/13/* *14 (SPL3/SPL11/SPL12/* *SPL13/SPL14)*	sRNA sequencing and experiment validation	Ge et al., 2018
*Osa-miR160f-5p*	*Oryza sativa*	*Auxin response factor 16 (ARF16)*	sRNA sequencing	Wu et al., 2017
*Osa-miR167a-5p*	*Oryza sativa*	*NB-ARC domain containing protein (NB-ARC)*	sRNA sequencing	Wu et al., 2017
*OsmiR396*	*Oryza sativa*	*Growth regulating factor 8 (OsGRF8)*	sRNA sequencing and experiment validation	Dai et al., 2019
*OsmiR159*	*Oryza sativa*	*OsGAMYBL2*	Experiment validation	Shen et al., 2023
*Osa-miR812s*	*Oryza sativa*	*Pectin methylesterase inhibitor (PEMI)*	sRNA sequencing	Nanda et al., 2020
*Osa-miR530-5p*	*Oryza sativa*	*Allene oxide synthase (AOS)*	sRNA sequencing	Nanda et al., 2020
*Osa-miR3980a-5p*	*Oryza sativa*	*Squamosa promoter binding protein (SBP)*	sRNA sequencing	Nanda et al., 2020
*Osa-miR156l-5p*	*Oryza sativa*	*No apical meristem (NAM)*	sRNA sequencing	Nanda et al., 2020
*Osa-miR2118g*	*Oryza sativa*	*NB-ARC domain containing protein (NB-ARC)*	sRNA sequencing	Nanda et al., 2020
*Osa-miR435*	*Oryza sativa*	*α/β hydrolase*	sRNA sequencing	Nanda et al., 2020
*Osa-miR2871a-3p*	*Oryza sativa*	*Glycosyltransferase family protein (GTF)*	sRNA sequencing	Nanda et al., 2020
*Osa-miR172a*	*Oryza sativa*	*AP2/EREBP family transcription factor (AP2/ERE)*	sRNA sequencing	Nanda et al., 2020
*Osa-miR156b-3p*	*Oryza sativa*	*GDSL-like lipase (GDSL)*	sRNA sequencing	Tan et al., 2020
*Osa-miR169i-5p.2*	*Oryza sativa*	*Leucine rich repeat family protein (LRR)*	sRNA sequencing	Tan et al., 2020
*Nlu-miR-14-3p*	*Nilaparvata lugens*	*NlInR* genes	sRNA sequencing	Xu et al., 2020
*Nlu-miR-9a-5p*	*Nilaparvata lugens*	*NlInR* genes	sRNA sequencing	Xu et al., 2020
*Nlu-miR-315-5p*	*Nilaparvata lugens*	*NlInR* genes	sRNA sequencing	Xu et al., 2020
*Nlu-miR-1000-1-3p*	*Nilaparvata lugens*	*Ultrabithorax (NlUbx)*	sRNA sequencing	Xu et al., 2020
*Nlu-mir-9a*	*Nilaparvata lugens*	*Ultrabithorax (NlUbx)*	Experiment validation	Li et al., 2021
*Nlu-miR-8-5p*	*Nilaparvata lugens*	*Membrane-bound trehalase (Tre-2)*	sRNA sequencing	Chen et al., 2013
*Nlu-miR-2a-3p*	*Nilaparvata lugens*	*Phosphoacetylglucosamine mutase (PAGM)*	sRNA sequencing	Chen et al., 2013
*Nlu-miR-4868b*	*Nilaparvata lugens*	*N. lugens glutamine synthetase (NlGS)*	sRNA sequencing and experiment validation	Fu et al., 2015
*Nlu-miR-173*	*Nilaparvata lugens*	*N. lugens Ftz-F1 (NlFtz-F1)*	sRNA sequencing and experiment validation	Chen et al., 2018
*Nlu-miR-2703*	*Nilaparvata lugens*	*N. lugens chitin synthase gene A*	Experiment validation	Li et al., 2017
*Nlu-miR-34-5p*	*Nilaparvata lugens*	*Hormone receptor 4 (HR4)/Caspase-1 (Cp-1) and Spermatogenesis-associated protein 20 (SPATA20)*	sRNA sequencing and experiment validation	Wang et al., 2022
*Osa-miR162a* ^a^	*Oryza sativa*	*N. lugens target of rapamycin (NlTOR)*	Conserved miRNA function prediction and experiment validation	Shen et al., 2021; Chen et al., 2023
*Osa-miR5795* ^a^	*Oryza sativa*	*N. lugens vitellogenin (NlVg)*	sRNA sequencing and experiment validation	Lü et al., 2022

a: Rice-derived sRNAs that function with cross-kingdom RNA interference to the brown planthopper.

It is well known that the phytohormone signaling plays an important role in rice defense against BPH (Zhou et al., 2009). Recent research suggests that miRNAs regulate rice resistance to BPH by post-transcriptionally regulating the expression of target genes involved in phytohormone signaling. For example, *Osa-miR156* negatively regulates BPH resistance by modulating jasmonic acid (JA) signaling (Ge et al., 2018). *Osa-miR156*-silenced plants (MIM156) exhibited increased resistance to BPH via upregulated expression of *OsMPK3* and *OsMPK6* and downregulated expression of *OsWRKY70*, a transcription factor which positively regulates JA signaling. Furthermore, the expression of the JA biosynthesis gene *OsHI-LOX* and the contents of JA and bioactive jasmonoyl-isoleucine (JA-Ile) were significantly reduced in MIM156 plants. Altogether, it appears that *Osa-miR156* regulates JA biosynthesis and BPH resistance via the MAPK cascade in rice. In addition, *Osa-miR162a* is strongly induced by BPH herbivory in rice seedlings (Chen et al., 2023). Functional verification indicated that *Osa-miR162a* regulates BPH resistance in rice by inhibiting the α-linolenic acid metabolism pathway, which itself regulates JA biosynthesis (Chen et al., 2023).

In rice, secondary metabolites have been shown to inhibit both the feeding and development of BPH. Furthermore, miRNAs can regulate the expression of genes involved in secondary metabolite biosynthesis to modulate BPH resistance. For example, *OsmiR396* was found to negatively regulate BPH resistance via the *OsmiR396–growth-regulating factor 8* (*OsGRF8*)*–OsF3H*–flavonoid module (Dai et al., 2019). Transgenic plants over-expressing *growth-regulating factor 8* (*OsGRF8*), the target gene of *OsmiR396*, exhibit enhanced BPH resistance due to downregulation of *OsmiR396*. Overall, it appears that *OsmiR396-OsGRF8* modulates BPH resistance by regulating the expression of the *flavanone 3-hydroxylase* (*OsF3H*) gene, which is involved in flavonoid biosynthesis (Dai et al., 2019). More recent research indicated that *OsmiR159* negatively regulates BPH resistance through the *OsmiR159*–*OsGA*-*MYBL2* module and the *OsmiR159*–*OsGAMYBL2–GS3* signaling pathway (Shen et al., 2023). Despite these advancements, the molecular mechanism underlying miRNA-mediated BPH resistance in rice is still poorly understood.

## The roles of sRNAs in BPH physiology

Advances in genomics have greatly expanded our understanding of the roles sRNAs play in BPH physiology and environmental response (Sattar and Thompson, 2016; Zha et al., 2016). Emerging evidence suggests that sRNAs participate in BPH metamorphosis, wing polyphenism, molting, and reproductive development (Chen et al., 2013; Xu et al., 2013; Chen et al., 2018; Ye et al., 2019; Xu et al., 2020; Li et al., 2021; Wang et al., 2022). Combing transcriptomic and genomic data, Xu et al. (2013) identified key genes involved in the BPH siRNA and miRNA pathways. RNAi knockdown of these genes severely affected BPH development and morphology, suggesting that siRNAs and miRNAs may play a crucial role in BPH development and metamorphosis (Xu et al., 2013).

In BPH, wing polyphenism is determined by environmental cues such as the nutritional status of host rice plants, population density, and photoperiod (Xu et al., 2020; Li et al., 2021). These environmental cues affect wing polyphenism by way of several complex regulatory pathways, including insulin/IGF-1 signaling (IIS), juvenile hormone (JH), and 20-hydroxyecdysone (20E) signaling (Xu et al., 2015). Research suggests that these signaling pathways are modulated by an array of miRNAs. For example, RNA sequencing of long wing (LW) and short wing (SW) BPH strains identified a complicated miRNA network which may modulate wing morphological plasticity in a growth-stage dependent manner (Xu et al., 2020). Three miRNAs (*Nlu-miR-14-3p*, *Nlu-miR-9a-5p*, and *Nlu-miR-315-5p*) have been confirmed to interact with *NlInR* genes, which are the part of IIS signaling pathway (Xu et al., 2020). In addition, *Nlu-miR-34* has been shown to modulate wing polyphenism by targeting *NlInR1* and mediating the cross-talk between the IIS, JH, and 20E signaling pathways via a positive autoregulatory feedback loop (Ye et al., 2019). Both Nlu-miR-1000-1-3p (Xu et al., 2020) and *Nlu-*mir-9a (Li et al., 2021) were predicted to target the wing development regulatory gene *Ultrabithorax* (*NlUbx*), and both were found to be differentially expressed between LW and SW BPH. Finally, the *NlInRs/Nlu-mir-9a/NlUbx* regulatory cascade appears to control wing dimorphism by regulating the host’s nutritional status (Li et al., 2021).

Molting is crucial to normal insect development, and is at least partially controlled by the chitin biosynthesis pathway and 20E signaling (Chen et al., 2013). Through deep miRNA sequencing of BPH instars at specific stages and during four molting periods, 21 (Chen et al., 2013) and 36 (Chen et al., 2018) specific mature miRNAs were identified, respectively. Among them, *Nlu*-*miR-8-5p*, *Nlu*-*miR-2a-3p*, and *Nlu*-*miR-173* were found to target genes in the chitin biosynthesis pathway, as well as transcription factor *NlFtz-F1*. All three miRNAs appear to regulate molting and chitin biosynthesis through 20E signaling (Chen et al., 2013; Chen et al., 2018). The expression of *chitin synthase gene A* was downregulated when its specific siRNA and its regulated miRNA (*Nlu*-*miR-2703*) were injected into BPH, reducing both chitin biosynthesis and molting success (Li et al., 2017).

sRNAs have also been found to regulate BPH fecundity by modulating the expression of genes associated with reproductive development. For example, injecting *Nlu-miR-34-5p* mimics can decrease BPH fecundity by reducing *vitellogenin (Vg*) expression (Wang et al., 2022). The biosynthesis of Vg is crucial for oocyte accumulation and successful reproduction (Wang et al., 2022). Glutamine synthetase (NlGS), a protein involved in ovary development which regulates Vg accumulation, has been identified as a target of *Nlu-miR-4868b* (Zhai et al., 2013; Fu et al., 2015). *NlGS* expression was downregulated following injection of the *Nlu-miR-4868b* mimic, but upregulated following injection of the *Nlu-miR-4868b* inhibitor. Additionally, overexpression of *Nlu-miR-4868b* reduced both insect fecundity and *Vg* expression.

Finally, miRNAs play important regulatory roles in environmental responses such as the adaptation to resistant rice varieties. Zha et al. (2016) constructed and sequenced two sRNA libraries using two BPH populations exhibiting different levels of virulence: biotype 1, which only survives on the susceptible rice variety ‘Taichung Native 1 (TN1)’, and biotype Y, which is able to survive on the resistant rice variety ‘YHY15’ (carrying BPH-resistance gene *Bph15*). The researchers identified 26 DEMs between these two BPH populations, suggesting that these BPH miRNAs may regulate adaptability to resistant rice varieties. However, the precise functions of these miRNAs require further confirmation.

## Cross-kingdom RNAi in the rice-BPH interaction

Research suggests that sRNAs can be transferred between host plants and interacting organisms, thereby inducing gene silencing via a mechanism known as “cross-kingdom RNAi” (Huang et al., 2019). This scenario was first reported in the interaction between plants and fungi. For example, gray mold (*Botrytis cinerea*)-derived sRNAs were found to be able to control the *Arabidopsis thaliana* RNAi system by binding to AGO1, ultimately silencing genes involved in plant immunity (Weiberg et al., 2013). Cross-kingdom RNAi has also been observed in the rice-BPH interaction (Shen et al., 2021; Lü et al., 2022). Rice-derived sRNAs may be ingested when BPH feed on rice plants, allowing them to regulate BPH gene expression.

Recently, rice-derived *Osa-miR162a*, a conserved plant miRNA, was found to effectively silence *NlTOR* (*Target of rapamycin*) expression in BPH through the cross-kingdom RNAi mechanism (Shen et al., 2021). Both ingestion and injection of *Osa-miR162a* mimics result in reduced female BPH fecundity and Vg activity, which is regulated by the TOR signaling pathway. In addition, allowing BPH adults to feed on *Osa-miR162a*- or *Osa-miR162a-m1* (a modified derivative of *Osa-miR162a*)-overexpressing transgenic rice lines consistently resulted in reduced egg production and hatching success. These results suggest that these miRNAs confer resistance to BPH in rice, and that *Osa-miR162a* may be a potential target for BPH control (Shen et al., 2021; Chen et al., 2023).

Another rice-derived miRNA, *Osa-miR5795*, has also been found to impact BPH fecundity (Lü et al., 2022). By sequencing and analyzing sRNAs from six rice varieties exhibiting variable BPH resistance, 45 resistance-related DEMs were identified between BPH-susceptible and BPH-resistant rice varieties prior to BPH infestation, as well as 144 feeding-induced DEMs. Twenty-five of these DEMs were shared between both groups and were found to be directly involved in the rice-BPH interaction. In addition, seven potential cross-kingdom miRNAs were identified, and their targets were primarily involved in fecundity, feeding, digestion, and detoxification. Based on their predicted binding sites, two of these cross-kingdom miRNAs were selected to verify their function in BPH fecundity. Consequently, BPH oviposition was significantly reduced following injection with *Osa-miR5795* mimics targeting the fecundity marker gene *NlVg* (Lü et al., 2022).

Both of these rice-derived miRNAs (*Osa-miR162a* and *Osa-miR5795*) appear to play an important role in rice-BPH interactions through cross-kingdom regulation of *NlTOR* and *NlVg* expression, both of which regulate fecundity in BPH (**Table 1**). However, to date no sRNAs, particularly BPH-derived miRNAs, appear to be involved in rice-BPH interactions through cross-kingdom RNAi trafficking.

## Application of cross-kingdom RNAi in crop protection

**Figure 1:**
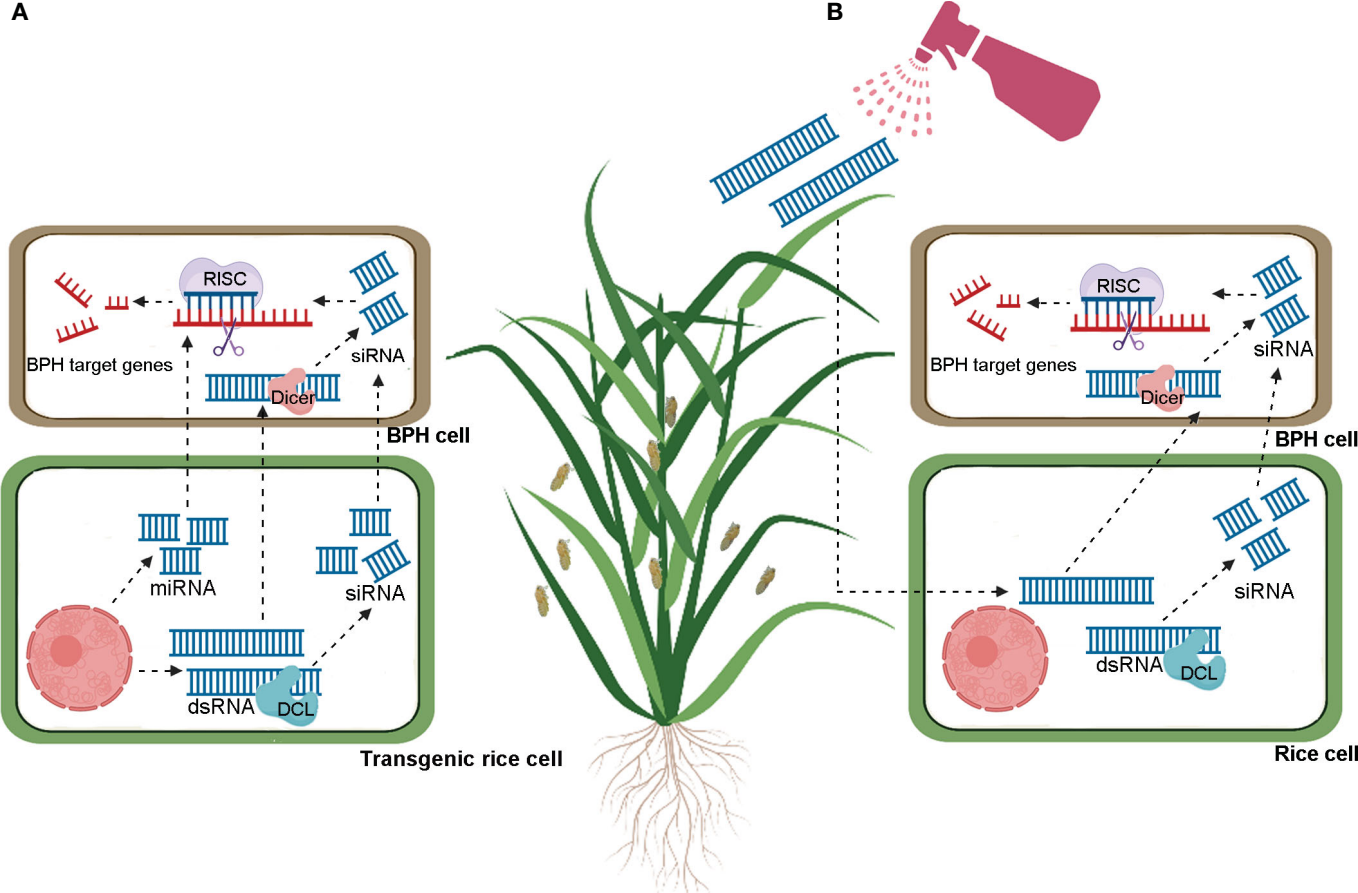
Schematic models for brown planthoppers control through host induced gene silencing (HIGS, A) and spray induced gene silencing (SIGS, B). In HIGS **(A)**, transgenic plants produce exogenous dsRNA or miRNA, or external spraying **(B)** delivers exogenous dsRNA. These dsRNA are processed into small interfering RNAs (siRNAs) by rice Dicer-like (DCL) proteins. The siRNAs are then transferred to brown planthopper (BPH) cells and bind to complementary sequences on BPH target mRNA. Through the assistance of the RNA-induced silencing complex (RISC), the target transcripts are silenced. Additionally, exogenous dsRNA and miRNAs produced by transgenic plants can be directly absorbed by BPH, resulting in gene silencing.

miRNA-mediated gene regulation has emerged as a novel strategy to improve insect resistance in crop plants, including rice. Host-induced gene silencing (HIGS) is a novel concept based on the cross-kingdom RNAi mechanism. HIGS involves overexpressing insect-targeted double-stranded RNAs (dsRNAs) or artificial miRNAs in host plants to specifically block the expression of feeding- and survival-related genes in target pests and pathogens (Huang et al., 2019; Jiang et al., 2023; Mahanty et al., 2023) (**Figure 1A**). A growing number of studies have demonstrated the successful application of HIGS in crop protection (Escobar et al., 2001; Seemanpillai et al., 2003; Zha et al., 2011; Van et al., 2014; Coleman et al., 2015; Shivakumara et al., 2017; Panwar et al., 2018). In this context, we will use the application of HIGS to manage BPH as an example. Two salivary proteins secreted by BPH are mucin-like protein (NlMLP) and salivary protein 1 (NlSP1). Ectopic expression of these genes in tobacco (*Nicotiana benthamiana*) leaves induced the expression of defense-related genes and callose deposition, suggesting that these two proteins function as elicitors (Shangguan et al., 2018; Huang et al., 2020). Compared to controls which received no injection or were injected with *dsGFP*, insects injected with *dsNlMLP* or *dsNlSP1* exhibited significantly reduced weight gain and survival rates, suggesting that NlMLP and NlSP1 are essential for BPH survival (Shangguan et al., 2018; Huang et al., 2020). Similarly, BPH feeding on transgenic plants constitutively expressing *dsNlMLP* or *dsNlSP1* also exhibited reduced weight gain and survival rates compared to insects feeding on wild type plants (Shangguan et al., 2018; Huang et al., 2020). Although allowing insects to feed on plants overexpressing exogenous dsRNA was not as effective as injecting insects directly, HIGS remains a promising pest control strategy. However, the implementation of HIGS depends on the generation of transgenic plants, which is both time-intensive and costly (Jiang et al., 2023; Mahanty et al., 2023). These limitations have so far hampered the application of HIGS to BPH control in rice.

Recently, a novel RNAi-based crop protection strategy called “spray-induced gene silencing (SIGS)” has been developed (Jiang et al., 2023; Mahanty et al., 2023). As the name implies, SIGS does not require genetic modification and instead involves simply spraying crop plants with synthesized exogenous dsRNA to selectively knock down insect or pathogen genes (**Figure 1B**). This technology has been successfully used to control rice blast disease (*Magnaporthe oryzae*) by spraying dsRNA targeting the fungal pathogenicity gene *MoDES1* (Sarkar and Roy-Barman, 2021). Recently, a nanocarrier-dsRNA spray delivery system was developed to control the white-backed planthopper (WBPH) (*Sogatella furcifera*) under laboratory conditions (Guo et al., 2023a, and Guo et al., 2023b). The results demonstrated the efficacy of the nanocarrier spray system for inducing RNAi-mediated knockdown of WBPH genes, including *SfTH*, *SfEGFR*, *Sfzfh-2*, *SfAbd-A*, and *SfAbd-B.* In addition, the treatment resulted in significant phenotypic defects and increased mortality in WBPH (Guo et al., 2023a, and Guo et al., 2023b). These promising results lay a foundation for the further development and application of SIGS to control rice pests, including BPH.

## Perspectives and challenges

A growing body of research has revealed the involvement of sRNAs in the interaction between rice and BPH. The majority of these sRNAs have been predicted and/or identified through multi-omics analyses, and their targets have been predicted computationally. However, many of these results still require experimental validation. Moreover, the molecular mechanisms underlying sRNA-mediated rice-BPH interactions remain poorly understood. The pathways of sRNA transfer between rice and BPH should also be comprehensively evaluated. Our growing understanding of cross-kingdom RNAi has paved the way for the development of promising agricultural pest control strategies, including HIGS and SIGS. Nevertheless, HIGS and SIGS face several technical challenges. The stability and uptake efficiency of dsRNA and sRNA need to be strengthened and off-target activities must be avoided. We predict that the development and application of environmentally-friendly RNAi-based technology will become an agronomic research focus, and that the communication of cross-kingdom sRNAs will emerge as a hot research topic.

## Author contributions

SJ: Project administration, Writing – original draft, Writing – review & editing. JX: Writing – review & editing. HT: Writing – review & editing. PL: Writing – review & editing. BY: Writing – original draft, Writing – review & editing. QL: Writing – original draft, Writing – review & editing.
